# Prevalence and Risk Factors of Kaposi's Sarcoma-Associated Herpesvirus Infection among Han and Uygur Populations in Xinjiang, China

**DOI:** 10.1155/2021/2555865

**Published:** 2021-12-31

**Authors:** Zhi Wen, Wenli Li, Yuan Fang, Chang Zhou, Kang Lin, Huanwu Wu, Yiting Zhang, Yulin Zhu, Xingchen Xu, Yan Zeng, Baojing Lu, Linding Wang

**Affiliations:** ^1^Department of Microbiology and Parasitology, The Key Laboratory of Microbiology and Parasitology of Anhui Province, The Key Laboratory of Zoonoses of High Institutions in Anhui, School of Basic Medical Sciences, Anhui Medical University, Hefei 230032, Anhui, China; ^2^The People's Hospital of Xuancheng City, Xuancheng 242000, Anhui, China; ^3^Anhui No. 2 Provincial People's Hospital, Hefei 230032, Anhui, China; ^4^Department of Pediatrics, The First Affiliated Hospital of Anhui Medical University, Hefei 230032, Anhui, China; ^5^Second Clinical Medical College, Anhui Medical University, Hefei 230032, Anhui, China; ^6^Key Laboratory of Xinjiang Endemic and Ethnic Disease and Department of Biochemistry, School of Medicine, Shihezi University, Shihezi 832000, Xinjiang, China

## Abstract

Kaposi's sarcoma-associated herpesvirus (KSHV) is the causative agent of Kaposi's sarcoma (KS), which is endangering human health worldwide, especially in Africa, Europe, the United States, and parts of Asia. The aim of this study was to investigate the prevalence of KSHV in Xinjiang. Three KSHV recombinant proteins (ORF65, ORF73, and K8.1) were used to detect KSHV infection. The serum samples to be tested were detected by an indirect ELISA method. The overall infection rate of KSHV in Xinjiang was 25.60%, with a higher infection rate in the Uygur population of 29.79%. After adjusting for possible confounders, Uygur (OR = 3.95, 95% CI 2.64–6.12, *P* < 0.001), agriculture and livestock (OR = 1.60, 95% CI 1.20–2.17, *P* = 0.002), age ≤ 50 years (OR = 1.50, 95% CI 1.13–2.00, *P* = 0.006), and predominantly meat-based diet (OR = 1.72, 95% CI 1.11–2.78, *P* = 0.018) were significantly associated with the odds of KSHV seropositivity correlation. Three unique sequences of KSHV were obtained in this study; genotypic analysis showed that the three unique sequences were all subtype A2.

## 1. Introduction

Kaposi's sarcoma-associated herpesvirus (KSHV), also known as human herpesvirus type 8 (HHV-8), [1, 2] belongs to the family of gamma herpesviruses. It was first discovered in 1994 by Chang and Moore in the tissues of Kaposi's sarcoma. [3] KSHV is also the etiology of primary effusion lymphoma (PEL) and multicentric Castleman's disease (MCD). [1, 4–6] Previous studies have shown that KSHV has a global distribution, mainly in Africa, with seroprevalence greater than 40%. [7, 8] KSHV seroprevalence is less than 10% in the Americas, [9, 10] whereas in parts of Asia and Europe, the seropositivity rate for KSHV is between 10% and 20%. [11, 12]

In China, the overall positivity rate for KSHV is between 10.00% and 16.80%, and the infection rates of KSHV in the general population in the vast majority of provinces is lower than 10.00%. [13–15] Xinjiang, located in northwest China, has a high prevalence of KSHV. Previous studies have shown that the prevalence of KSHV infection in the general population in Xinjiang is between 19.20% and 35.40%. [16, 17] The unique lifestyle and cultural differences of the Uygur population [18, 19] may promote the spread of KSHV and consequently KSHV infection.

KSHV is known to be associated with four types of KS, including classical KS, local KS, transplanted KS, and AIDS-related KS. [12] The K1 gene is located at the right end of the KSHV genome, and the K1 protein has two extracellular domains that are extremely variable, with a difference of up to 60% at the amino acid level. [20] According to the polymorphism of K1 gene, KSHV is mainly classified into A, B, C, D, E, and F subtypes. [20–22] Among them, the subtype A of KSHV was most commonly found in North America and was mainly prevalent in patients with typical KS; subtype B was unique to Africa; subtype C was mainly distributed in the Mediterranean, Middle East and Asia; subtype D was characteristic of people from Japan, Southeast Asia, and some Pacific islands; and subtype E was mainly distributed in Brazil of South America. [23–25] The geographical clustering of KSHV appeared to be linked to the movements of ancient infected populations. [26]

However, not much is known about KSHV infection in Xinjiang. Xinjiang is inhabited by the Uygur and Han populations, and the risk factors for KSHV in the region are also not well understood, and the potential risk factors for KSHV infection have not been fully assessed. The aim of this study was to investigate the seroprevalence of KSHV among different ethnic groups in the region and to assess the relationship between KSHV prevalence and the relationship between demographic factors and everyday behavior.

## 2. Materials and Methods

### 2.1. Expression and Purification of Target Proteins

The C-terminal of ORF65, the C-terminal of ORF73, and K8.1*γ* were selected as the target genes [5, 27, 28]. All three recombinant plasmids were constructed previously [16]; recombinant plasmids containing the target genes were transformed to *E. coli* BL21 (DE3). Proteins were expressed with induction of IPTG at a concentration of 1 mM. The resulting target proteins were collected after lysozyme treatment and ultrasonic fragmentation. The target proteins were purified using nickel column affinity chromatography to obtain a purified viral protein monomer.

### 2.2. Establishment of an Indirect ELISA Assay

The concentration of antigen and the dilution of serum antibodies in the ELISA indirect method were determined using the chessboard method. [16] Each of the three antigens was encapsulated in 96 wells with the appropriate concentration. The sera, the horseradish peroxidase (HRP)–labeled sheep anti-human IgG, the substrate tetramethylbenzidine (TMB), and termination solution were added in succession. The absorbance value (OD) was measured at 450 nm.

### 2.3. Sample Collection

This study was conducted in the Shihezi prefecture in Xinjiang, China, from the beginning of June to the end of August in 2019. Participants were recruited using the multistage sampling method. Participates with no age restriction, no clinical symptoms for the last 6 months, and living in Xinjiang province were eligible for enrollment. Participants were asked to complete a detailed questionnaire, which included name, gender, age, education, exercise, dietary preferences, and so on. A total of 1078 sera were tested in this study, and all test samples were provided by Professor Yan Zeng from the School of Medicine, Shihezi University. The sera to be tested were stored at −80°C in a refrigerator, all samples were from the First Affiliated Hospital of Shihezi University, and all samples were assigned to a unique number.

Permission to conduct the study and informed written consents were obtained in accordance with a protocol approved by the Biomedical Ethics Committee of the Anhui Medical University.

### 2.4. Serological Testing

Three target proteins were used as antigens to coat the three 96-well plates to detect OD values in the sera of known KSHV-negative patients, and standard deviation (SD) values were obtained for each target protein based on OD values. Each serum to be tested was subjected to an ELISA for each of the three target proteins, with detection based on positive wells, negative wells, and blank wells. The cut-off value is the mean OD value of the negative control well plus 5 times standard deviation (SD). In all tests, serum S945 from AIDS-KS patients and serum H475 from healthy blood donors were used as positive and negative controls, respectively, all of which were stored in our laboratory, and the blank control was PBS. Each sample was also tested three times. If the OD value of any of the three tests is above the cut-off value, the sample is determined to be a KSHV-positive sample. [16]

### 2.5. Statistical Analysis

SPSS 16.0 software was used to analyze the different independent factors of KSHV seroprevalence. *P* values were introduced when assessing hazard ratios, and *P* < 0.05 is considered a statistically significant difference; 95% CI was used to show the significant differences. CI is calculated on the basis of logistic regression coefficients and standard deviations.

### 2.6. Genotyping of KSHV from Seropositive Samples

The DNA of peripheral blood mononuclear cells from KSHV serum-positive patients was extracted by QIAamp MinElute Virus Spin Kit, and the experimental procedures were strictly followed by the instructions. The VR1 region of K1 of KSHV was amplified by nested PCR, and the amplified product size was 455 bp. [16] Clustal W was used for sequence alignment between the three unique sequences (The GenBank Accession Number are MZ490465, MZ490466, and MZ490467), and 22 KSHV strains were obtained from GenBank. MEGA (version 7.0) was used to construct the phylogenetic tree by neighbor-joining analysis. 1000 bootstrap samples were used to evaluate the statistical reliability of phylogenetic tree. Twenty-two strains of KSHV obtained from GenBank were composed of six strains of subtype A, five strains of subtype B, five strains of subtype C, four strains of subtype D, and two strains of subtype E. [16, 29]

## 3. Results

### 3.1. Expression and Purification of Target Proteins

Three viral proteins, ORF65, ORF73, and K8.1, were expressed. The three gene sizes are 270 bp, 651 bp, and 537 bp, respectively. The three genes were inserted into the expression vector pQE-80L. The molecular weights of the three expressed proteins were 9 kDa, 27 kDa, and 25 kDa, respectively. Purified proteins were achieved by using Ni^2+^ column affinity chromatography. Then, 96-well plates were used to detect antibodies to the virus in serum samples based on an ELISA method with these proteins. [16]

### 3.2. Characteristics of the Study Population

A total of 1078 serum samples (Han Chinese: 289, Uygur: 789) were investigated; the basic characteristics of the subjects are summarized in Table 1. There was no difference in marital status, gender, sugar addiction, diabetes, hepatitis, genetic history, disability, predominantly vegetarian diet, and so on between the two ethnic groups. Han patients were relatively more educated (*P* < 0.001), older (*P* < 0.001), more likely to drink alcohol (*P* < 0.001) or smoke (*P* = 0.023) compared to Uygur patients. In contrast, Uygur serum sample patients were more likely to be engaged in farming and animal husbandry (*P* < 0.001) and to have a more dietary preference for predominantly meat-based diets (*P* < 0.001).

### 3.3. Seropositivity Rates for KSHV Infection

The combined sensitivity of serological tests was 100%, and the specificity was 96% in previous studies. [13, 16] Of the 1078 serum samples, a total of 276 were identified as KSHV seropositive, a positivity rate of 25.60%, of which 28 (9.69%) were Han patients and 235 (29.79%) were Uygur patients. There were 97 (22.86%) male patients compared to 167 (25.54%) female patients. As shown in Table 2, the distribution of positive patients in terms of age group was mainly concentrated in the age group of 20 to 69 years with a positive rate of more than 20%, while the positive rate in the age group of 20 to 39 years was over 30%.

### 3.4. Analysis of Risk Factors

Univariate and multivariate logistic regression analyses were performed on the sample data, as shown in Tables 3 and 4. KSHV positivity was not related to literacy, marital status, sex, smoking, or alcohol consumption. KSHV seropositivity was associated with Uygur ethnicity (OR = 3.95, 95% CI 2.64–6.12, *P* < 0.001), working in agriculture and livestock (OR = 1.60, 95% CI 1.20–2.17, *P* = 0.002), and age ≤ 50 years (OR = 1.50, 95% CI 1.13–2.00, *P* = 0.006). KSHV seropositivity was found to have a significant correlation with dietary habits. Subjects with predominantly meat-based diet (OR = 1.72, 95% CI 1.11–2.78, *P* = 0.018) were more likely to have KSHV infection compared with vegetarian diet. From the multivariate logistic regression analysis, it was also clear that KSHV seropositivity was associated with ethnicity (OR = 4.01, 95% CI 2.51–6.66, *P* < 0.001) and meat-based diet (OR = 3.85, 95% CI 1.59–11.11, *P* = 0.006). However, the correlation with greasy eating habit, age, and centripetal obesity was not very significant, which might be influenced by sample size and confounding factors.

### 3.5. Subtype Analysis in Xinjiang

**Figure 1:**
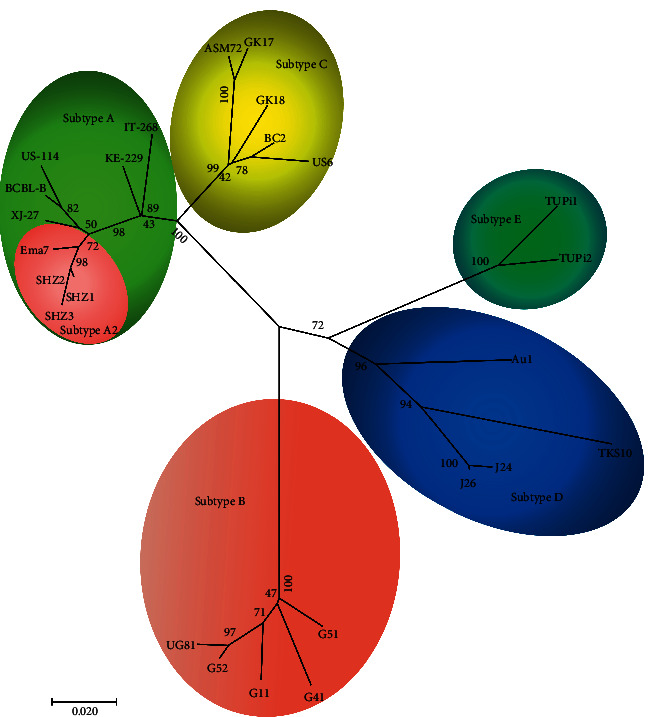
Phylogenetic tree was constructed based on the KSHV K1 sequence. Relationship of KSHV isolates in present study and isolates in the literature is shown. Branch lengths are drawn to scale, with the bar indicating 0.02 nt replacement per site. The isolates in this study were named SHZs; sequences of other KSHV isolates were obtained from NCBI. According to the phylogenetic tree, it can be concluded that three unique sequences belong to subtype A2 like Ema7.

Fifty positive samples were randomly selected, and DNA of their peripheral blood mononuclear cells was extracted. Three unique sequences were amplified by nested PCR and named SHZ1, SHZ2, and SHZ3, respectively. According to the phylogenetic tree drawn by MEGA, SHZ1, SHZ2, and SHZ3 were all subtype A2 (Figure 1).

## 4. Discussion

KSHV belongs to the gamma herpesvirus group and is a double-stranded DNA virus. The structure is the same as that of a common herpesvirus, with the innermost layer being genetic material, the middle layer being an icosahedral protein coat and the outermost layer being lipid. The double-layered outer membrane is also embedded with various glycoproteins. [30–32] These glycoproteins play an important role in the invasion of viruses into host cells. [33–35] KSHV genome sequences were found in all types of KS; independent epidemiological studies have shown a strong association between KS and KSHV infection. [3, 36–38] In Xinjiang, China, one or more patients were diagnosed with KS every year, suggesting a high incidence of KS in this region; PCR and immunohistochemical results showed that all KS cases were associated with KSHV infection. [39] Another study in Xinjiang has found that the KSHV DNA was detected in 17 of 20 KS tissues (85%), while the KSHV DNA was not detected in 20 non-KS tissues. [40] The aforementioned studies have shown that KSHV plays an important role in the occurrence of KS and may be involved in the pathogenesis of KS, but it is not the only cause of its occurrence.

In this study, we chose an indirect ELISA method for the detection of antibodies to KSHV in the samples. There are three reasons why we use ELISA to detect KSHV in sera. First, the overall cost of the method is low, which is suitable for serological testing of large numbers of samples; second, the method has good accuracy and repeatability, which facilitates the detection of indeterminate samples; [16] third, the method requires relatively low experimental condition and does not require the use of high-precision instruments and equipment [41, 42].

The Xinjiang Autonomous Region, located in northwest China, is a multiethnic region with a total of about 13 ethnic minorities and Han Chinese. There are different living habits, food styles, and religious beliefs among them. [16] Among the ethnic groups, the majority of the population is Han (about 40%), and among the minority groups, the Uygur population is the largest (45%), and there are also other minorities including the Hui, Kazakhs, and so on.

The results showed that the KSHV seropositivity rate of Uygur in this region was significantly higher than that of Han Chinese in the same region, and this ethnic difference might be due to different genetic backgrounds. This result is also consistent with the results of previous studies [43, 44]. In terms of occupational choice, people working in agriculture and pastoralism are more likely to be diagnosed as KSHV-positive, it could be that people who work in agriculture and pastoralism have poor sanitary conditions, suggesting that the agriculture and pastoralism is a predisposing factor for KSHV infection. In terms of diet, we found that people with meat-based diet might be more susceptible to infection than those who ate a balanced meat and vegetable diet; it might be because of saliva transmission of KSHV, [45] according to the unique dietary habits of Xinjiang, there might be parallel infection caused by sharing the same meat (such as roast whole sheep, etc.) and sharing the same tableware among many people, suggesting that the proper diet might be effective in reducing KSHV infection. We also found that the rate of KSHV seropositivity was significantly higher in people younger than 50 years than in those older than 50 years; it suggests that people younger than 50 are more susceptible to KSHV. No differences were found in KSHV infection in terms of sex, marital status, smoking, and alcohol consumption. [46, 47] The result is possibly influenced by sample content and other confounding factors.

In this study, more than a thousand serum samples were detected for KSHV infection using an ELISA method. The prevalence characteristics and susceptibility factors of the region were analyzed. However, more in-depth studies are lacking. For example, the amount of meat or vegetarian food consumed daily by the population of the region was not considered in analysis, and the genetic or underlying diseases were also not considered. All these factors have the potential to influence KSHV infection. Nonetheless, this study contributes to a better understanding of the prevalence of KSHV in the region's population.

It is common that the human population comigrate with viruses. KSHV subtypes A and C are most seen in the Mediterranean, Middle Eastern, and Asian regions, [23] which were parts of the ancient Great Silk Road. KSHV subtypes C and A have been reported in Xinjiang before. [16, 29] In this study, only subtype A was identified, probably due to the limited positive samples. From these data, KSHV could be further speculated to have spread along the Silk Road with the human population.

In conclusion, the overall prevalence of KSHV infection in Xinjiang is higher than that of the rest of China, and the KSHV prevalence in Uygur population is higher than that in Han Chinese living in the same area. It is worth noting that the cultural and culinary exchanges between different ethnic groups may facilitate the interethnic transmission of KSHV. The results of this study will provide a scientific basis for the prevention and control of KSHV infection in the region.

## Figures and Tables

**Table 1:** Characteristics of Han and Uygur research participants in the Xinjiang region.

	Total, *n* = 1078, *n* (%)	Han ethnic group, *n* = 289, *n* (%)	Uygur ethnic group, *n* = 789, *n* (%)	*F*/*χ*^2^	*P-*value
Education level					
Primary school and below	650 (60.3)	109 (37.72)	541 (68.57)	71.669	<0.001
Primary and above	353 (32.75)	146 (50.52)	207 (26.24)		

Occupation					
Agriculture and animal husbandry	657 (60.95)	122 (42.22)	535 (67.81)	56.363	<0.001
Others	420 (38.96)	166 (57.45)	254 (32.19)		

Marital status					
Unmarried	22 (2.04)	5 (1.73)	17 (2.15)	0.038	0.845
Married/divorced/bereaved of one's spouse	1054 (97.77)	284 (98.28)	771 (97.72)		

Genders					
Male	420 (38.96)	113 (39.1)	307 (38.91)	0.009	0.926
Female	654 (60.67)	173 (59.86)	481 (60.96)		

Age	47.42 ± 11.48	50.53 ± 9.64	46.28 ± 11.89	6.001	<0.001
≤50	626 (58.07)	129 (44.64)	497 (62.99)	28.77	<0.001
>50	451 (41.84)	160 (55.36)	291 (36.88)		

Smoking					
Never	892 (82.75)	225 (77.85)	667 (84.54)	5.172	0.023
Yes	184 (17.07)	62 (21.45)	122 (15.46)		

Drinking alcohol					
no	963 (89.33)	221 (76.47)	742 (94.04)	65.499	<0.001
yes	109 (10.12)	65 (22.49)	44 (5.58)		

Meat and vegetable balance					
No	917 (85.06)	210 (72.66)	707 (89.61)	0.019	0.878
Yes	151 (14.01)	77 (26.64)	74 (9.38)		

Meat-based diet					
No	59 (5.47)	17 (5.88)	42 (5.32)	41.667	<0.001
Yes	1000 (92.76)	270 (93.43)	730 (92.52)		

Vegetarian diet					
No	95 (8.81)	57 (19.72)	38 (4.82)	55.705	<0.001
Yes	967 (89.7)	230 (79.58)	737 (93.41)		

Glycosophilic					
No	73 (6.77)	14 (4.84)	59 (7.48)	2.066	0.151
Yes	987 (91.56)	273 (94.46)	714 (90.49)		

Diabetes					
No	958 (88.87)	255 (88.24)	703 (89.1)	0.804	0.370
Yes	85 (7.88)	27 (9.34)	58 (7.35)		

Hepatitis					
No	997 (92.49)	271 (93.77)	726 (92.02)	0.101	0.750
Yes	46 (4.27)	11 (3.81)	35 (4.44)		

Genetic history					
No	857 (79.5)	257 (88.93)	600 (76.05)	0.697	0.404
Yes	43 (3.99)	16 (5.54)	27 (3.42)		

Disabled					
No	874 (81.08)	265 (91.7)	609 (77.19)	0.003	0.955
Yes	21 (1.95)	7 (2.42)	14 (1.77)		

**Table 2:** Age distribution of positive patients.

Age groups	Numbers *N* (%)	Number of positives *N* (%)
<19	61 (5.66)	6 (9.84)
20∼29	69 (6.40)	24 (34.78)
30∼39	204 (18.92)	62 (30.39)
40∼49	303 (28.10)	79 (26.07)
50∼59	298 (27.64)	72 (24.16)
60∼69	129 (11.96)	32 (24.80)
>70	14 (1.30)	1 (7.14)

**Table 3:** Univariate logistic regression analysis.

	Number of positives, *n* (%)	OR (95% CI)	*P-*value
Nationality			
Han ethnic group	28 (9.69)		
Uygur ethnic group	235 (29.79)	3.95 (2.64–6.12)	<0.001

Education level			
Primary school and below	174 (26.77)		
Primary and above	73 (20.68)	0.95 (0.52–1.79)	0.856

Occupation			
Agriculture and animal husbandry	182 (27.7)	1.60 (1.20–2.17)	0.002
Others	81 (19.29)		

Marital status			
Unmarried	5 (22.73)		
Married/divorced/bereaved of one's spouse	258 (24.48)	0.97 (0.66–1.38)	0.873

Genders			
Male	96 (22.86)		
Female	167 (25.54)	1.16 (0.87–1.55)	0.319

Age			
≤50	172 (27.48)	1.50 (1.13–2.00)	0.006
>50	91 (20.18)		

Smoking			
Never	219 (24.55)		
Yes	44 (23.91)	0.97 (0.66–1.39)	0.854

Drinking alcohol			
No	242 (25.13)		
Yes	21 (19.27)	0.71 (0.42–1.15)	0.179

Meat and vegetable balance			
No	234 (25.52)		
Yes	25 (16.56)	0.93 (0.55–1.52)	0.77

Meat-based diet			
No	8 (13.56)		
Yes	249 (24.9)	1.72 (1.11–2.78)	0.018

Vegetarian diet			
No	22 (23.16)		
Yes	237 (24.51)	0.47 (0.21–0.95)	0.053

Oily			
No	22 (27.85)		
Yes	235 (23.98)	0.82 (0.50–1.39)	0.441

Waist–hip ratio			
Normal	73 (21.10)		
Centripetal obesity	172 (26.38)	1.34 (0.98–1.84)	0.066

**Table 4:** Multivariate logistic regression analysis.

	OR (95%)	*P-*value
Uygur ethnic group	4.01 (2.51–6.66)	<0.001
Meat-based diet	3.85 (1.59–11.11)	0.006
Oily	0.58 (0.30–1.15)	0.110
>50 years old	0.73 (0.51–1.05)	0.093
Centripetal obesity	1.32 (0.91–1.93)	0.152
Drinking alcohol	1.25 (0.71–2.13)	0.430

## Data Availability

The data that support the findings of this study are available from the corresponding author upon reasonable request.
